# Copy number variant and runs of homozygosity detection by microarrays enabled more precise molecular diagnoses in 11,020 clinical exome cases

**DOI:** 10.1186/s13073-019-0639-5

**Published:** 2019-05-17

**Authors:** Avinash V. Dharmadhikari, Rajarshi Ghosh, Bo Yuan, Pengfei Liu, Hongzheng Dai, Sami Al Masri, Jennifer Scull, Jennifer E. Posey, Allen H. Jiang, Weimin He, Francesco Vetrini, Alicia A. Braxton, Patricia Ward, Theodore Chiang, Chunjing Qu, Shen Gu, Chad A. Shaw, Janice L. Smith, Seema Lalani, Pawel Stankiewicz, Sau-Wai Cheung, Carlos A. Bacino, Ankita Patel, Amy M. Breman, Xia Wang, Linyan Meng, Rui Xiao, Fan Xia, Donna Muzny, Richard A. Gibbs, Arthur L. Beaudet, Christine M. Eng, James R. Lupski, Yaping Yang, Weimin Bi

**Affiliations:** 1 Baylor Genetics Laboratories, Houston, TX USA; 2 Department of Molecular and Human Genetics, Baylor College of Medicine, One Baylor Plaza, Houston, TX 77030-3411 USA; 3 Glenda Dawson High School, Pearland, TX USA; 4 Human Genome Sequencing Center, Baylor College of Medicine, Houston, TX USA; 5 Department of Pediatrics, Baylor College of Medicine, Houston, TX USA; 6 Texas Children’s Hospital, Houston, TX USA

**Keywords:** Exome sequencing, Microarray, Structural variation, Uniparental disomy, ROH, Dual molecular diagnoses, Exonic CNV in AR disorders

## Abstract

**Background:**

Exome sequencing (ES) has been successfully applied in clinical detection of single nucleotide variants (SNVs) and small indels. However, identification of copy number variants (CNVs) using ES data remains challenging. The purpose of this study is to understand the contribution of CNVs and copy neutral runs of homozygosity (ROH) in molecular diagnosis of patients referred for ES.

**Methods:**

In a cohort of 11,020 consecutive ES patients, an Illumina SNP array analysis interrogating mostly coding SNPs was performed as a quality control (QC) measurement and for CNV/ROH detection. Among these patients, clinical chromosomal microarray analysis (CMA) was performed at Baylor Genetics (BG) on 3229 patients, either before, concurrently, or after ES. We retrospectively analyzed the findings from CMA and the QC array.

**Results:**

The QC array can detect ~ 70% of pathogenic/likely pathogenic CNVs (PCNVs) detectable by CMA. Out of the 11,020 ES cases, the QC array identified PCNVs in 327 patients and uniparental disomy (UPD) disorder-related ROH in 10 patients. The overall PCNV/UPD detection rate was 5.9% in the 3229 ES patients who also had CMA at BG; PCNV/UPD detection rate was higher in concurrent ES and CMA than in ES with prior CMA (7.2% vs 4.6%). The PCNVs/UPD contributed to the molecular diagnoses in 17.4% (189/1089) of molecularly diagnosed ES cases with CMA and were estimated to contribute in 10.6% of all molecularly diagnosed ES cases. Dual diagnoses with both PCNVs and SNVs were detected in 38 patients. PCNVs affecting single recessive disorder genes in a compound heterozygous state with SNVs were detected in 4 patients, and homozygous deletions (mostly exonic deletions) were detected in 17 patients. A higher PCNV detection rate was observed for patients with syndromic phenotypes and/or cardiovascular abnormalities.

**Conclusions:**

Our clinical genomics study demonstrates that detection of PCNV/UPD through the QC array or CMA increases ES diagnostic rate, provides more precise molecular diagnosis for dominant as well as recessive traits, and enables more complete genetic diagnoses in patients with dual or multiple molecular diagnoses. Concurrent ES and CMA using an array with exonic coverage for disease genes enables most effective detection of both CNVs and SNVs and therefore is recommended especially in time-sensitive clinical situations.

**Electronic supplementary material:**

The online version of this article (10.1186/s13073-019-0639-5) contains supplementary material, which is available to authorized users.

## Background

Copy number variants (CNVs), ranging in size from 50 to 100 bp to several megabases, are the direct cause of genomic disorders and are also an underlying contributing genetic factor in both dominant or recessive human diseases, as well as complex traits [1–5]. Chromosomal microarray analysis (CMA) by either array comparative genomic hybridization or SNP arrays is the first-tier clinical testing for genome-wide detection of CNVs in pediatric patients with neurodevelopmental problems such as developmental/intellectual disabilities (DD/ID) and multiple congenital anomalies [6]. The resolution of CMA has increased during the last decade, enabling detection of CNVs from a few hundred kilobases to intragenic changes involving one or a few exons for known or candidate disease genes [7–10]. A meta-analysis in 2010 reported 15–20% diagnostic yield by CMA [6]. More recent studies suggest that the detection rate for pathogenic CNVs is ~ 10% for clinical CMA with CNV interpretation based on the American College of Medical Genetics and Genomics (ACMG) criteria [11]. Abnormal findings were reported in 14% of patients in a study using an Agilent SNP array with exon-targeted coverage for > 1860 genes [10]. In another study using an ultrahigh-resolution microarray in 5487 patients, overall CNV diagnostic yield was as high as 29.4%, but 9.2% were pathogenic findings whereas 20.2% were variants of unknown significance; the most frequent pathogenic CNV is the 15q11.2 BP1-BP2 deletion detected in 31 patients [12]. However, the association of the 15q11.2 deletion with neurodevelopmental clinical phenotypes is weak and of low penetrance; the clinical significance of this deletion is still being debated [13]. The pathogenic CNV detection rate of this study would drop to 8.6% if this deletion is classified as being of unknown significance.

Exome sequencing (ES), detecting single nucleotide variants (SNVs) and small indels (< 50–100 bp) in coding regions of the genome, has been increasingly applied for molecular diagnosis in clinical settings [14]. When used in a primarily pediatric patient cohort, ES yields a molecular diagnostic rate of approximately 25% [15, 16]. The diagnostic rate increases to 36.7% in trio analysis for a range of medical conditions presenting in the neonatal intensive care unit (NICU) [17]. Detection of homozygous and hemizygous single exon intragenic CNVs from ES can be readily achieved by algorithms such as HMZDelFinder [18]. Large genomic intervals with the absence of heterozygosity (AOH), potentially representing runs of homozygosity (ROH) regions and evidence for identity-by-descent (IBD), can also be detected through non-phased ES data by algorithms such as BafCalculator for potential uniparental disomy (UPD) or as regions of potential IBD when parental consanguinity and/or population substructure is present [19, 20]. However, the detection of heterozygous CNVs from ES data remains challenging; the exome capture procedure in ES can produce biases in the extent of capture of some individual exons, particularly GC-rich first exons, and result in an uneven distribution of reads in exonic regions [21]. Moreover, false negative and false positive rates vary greatly depending on the CNV detection algorithms employed [22–25]. Currently, microarray analysis remains the gold standard for the clinical detection of rare PCNVs.

Given the technical challenges to detect clinically relevant rare CNVs by ES, concurrent CMA and ES testing or sequential testing has been successfully applied to detect both SNVs and CNVs, enabling more precise molecular diagnosis for dominant as well as recessive disease traits [16, 26]. Many patients referred for ES have prior CMA studies, but CMA is either negative or CMA findings do not fully explain the clinically observed features. However, in a large portion of ES patients, the role of CNVs remains unknown, particularly for those < 50 kb in size. A large clinical cohort study is necessary to estimate the contribution of CNVs to the molecular diagnoses in patients referred for ES.

Here, we explored the contribution of copy number analysis in molecular diagnosis of ES patients through retrospective analysis of CNV/ROH identified by an Illumina SNP on 11,020 ES patients and by CMA on 3229 ES patients.

## Methods

### Clinical samples

This retrospective query included a total of 11,020 consecutive patients, including affected siblings but not including parents, studied by ES as a clinical service at Baylor Genetics (BG) from October 2011 to November 2017, 3229 of whom had CMA performed at BG. This analysis of aggregate clinical genomics data was approved by the Baylor College of Medicine Institutional Review Board (protocol H-37568).

### Exome sequencing

ES, previously referred to as whole-exome sequencing (WES) [27], was performed at BG for families by proband only or trio-ES (proband + biological parents confirmed by identity testing). The majority of subjects were pediatric patients with primarily neurological phenotypes or congenital anomalies. Prenatal ES cases were excluded from this study. This test targets approximately 20,000 genes to a mean coverage of greater than 130× with 95% of targeted regions covered at > 20×. Rare variants were filtered and annotated as described [15, 16]. In addition, homozygous and hemizygous deletions were detected from ES data using normalization of exome read depth as previously described [28, 29]. However, un-phased ES data were not applied for AOH/ROH detection in this cohort.

### Detection of copy number variations using the quality control (QC) arrays

An Illumina SNP array was run concurrently with a split DNA sample for each ES case as a QC measurement. Sample identity was confirmed when SNP array variant calls were concordant with ES variant data by next-generation sequencing. The first approximately 250 cases were run on Human1M-Duo array. HumanExome-12 array was used between 2012 and 2016, and the SNP array was switched to Infinium CoreExome-24 in May 2016. The HumanExome-12 array contains > 240k SNP markers in exonic regions (i.e., coding SNPs or cSNPs). Additional 307k SNPs from the Infinium Core-24 BeadChip were present in the CoreExome-24 array which contains ~ 268k exonic markers and ~ 152k intronic markers.

The QC array enables robust detection of ROH > 5 Mb throughout the genome and provides more precise intervals of ROH than does CMA using Agilent SNP array (Additional file 1: Figure S1). In addition, approximately 70% of PCNVs detectable by a clinical array were detected by the QC array (Additional file 2, Additional file 3); therefore, the QC array data were also analyzed for CNVs and ROH using cnvPartition 3.1.6 in Illumina GenomeStudio software to provide additional molecular diagnoses. CNVs were interpreted based on the ACMG guidelines [11], and only pathogenic or likely pathogenic CNVs and potential UPD-associated ROH, i.e., PCNVs/UPD, were included in clinical reports. Because the QC array was not designed for clinical detection of copy number changes, confirmatory testing using a clinical copy number assay was recommended. Comparing the results of confirmatory studies performed at BG indicated that all the reported findings from the QC array were confirmed by CMA.

### Chromosomal microarray analysis

A subset of ES samples also had CMA testing that was performed at BG as a clinical copy number test and conducted as described before [7, 10]. For the vast majority of cases, CMA was performed using an Agilent custom oligo array V6 to V11, while 19 cases had a BAC array and 47 cases had Illumina 1 M SNP array or Affymetrix Cytoscan analyses. CMA in about two thirds of cases was performed using custom-designed Agilent microarrays (v9, v10, or v11 arrays) with exonic coverage of > 4200 targeted disease or disease-candidate genes and “backbone” genome-wide coverage of one interrogating oligonucleotide/30 kb [8, 30]. These microarrays also contain 60k SNP probes which enable screening of > 10 Mb ROH.

CNVs were classified as pathogenic, likely pathogenic, variants of unknown clinical significance, likely benign, and benign based on the ACMG guidelines [11]. Pathogenic and likely pathogenic CNVs (PCNVs) include CNVs associated with well-established syndromes, de novo variants, and large microscopic changes. CNVs with evidence from either public or internal databases to support that the CNVs likely represent normal population variants were not considered as PCNVs even if they were de novo. In this study, the following changes were not classified as PCNVs due to insufficient evidence: *TMLHE* deletion, 15q11.2 BP1-BP2 deletion, heterozygous CNVs within the *CNTNAP2*, *A2BP1*, or *CNTN4* gene, a small duplication involving one end of *CHRNA7*, and *STS* duplication.

### Human phenotype ontology (HPO) term analysis

A subset of the ES cases with CMA performed at BG (*N* = 2876) was each annotated with HPO terms obtained from the clinical notes. Lower level HPO terms were mapped to the corresponding highest branch HPO term in the ontology under “Phenotypic abnormality” (HP:0000118), each of which covers clinical features associated with a distinct organ system. Logistic regression analysis of the cases with or without PCNV/UPD was performed to identify the variables (the number of unique top-level terms, age, and sex) that influence the PCNV/UPD detection rate. All statistical tests were performed in the R computing environment.

## Results

### CNV/ROH findings from the QC array in 11,020 ES cases

**Fig. 1:**
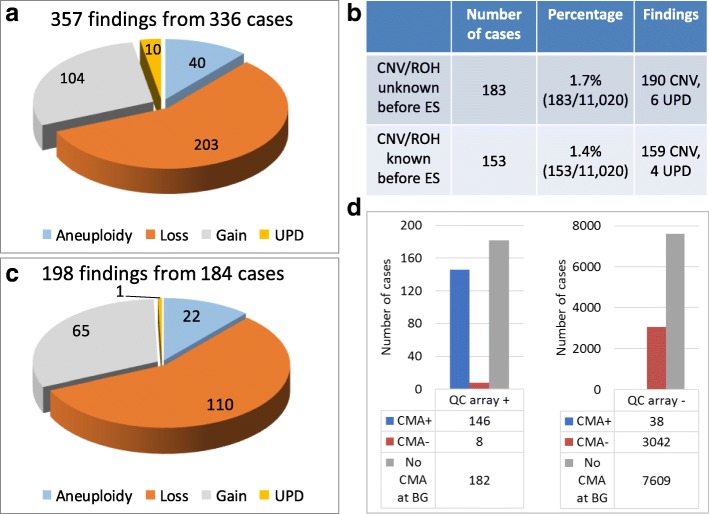
Summary of PCNV/UPD findings from the quality control array and CMA. **a** A pie chart to show the types of aberrations detected by the QC array. **b** A table to show the proportion of patients with PCNVs/UPD from the QC array that were known or unknown prior to ES testing. **c** A pie chart to show the types of aberrations detected by CMA. **d** A chart to correlate the findings from the QC array and those from CMA. “+” means with PCNV/UPD from the QC array or CMA; “-” means without PCNV/UPD findings

Concurrent QC array analysis was performed on all of the 11,020 consecutive clinical ES cases included in this study. PCNVs/UPD (*N* = 357) were identified in 336 (3%) patients by the QC array (Fig. 1a). PCNVs (*N* = 347) were detected in 327 patients, including 40 aneuploidies, 203 deletions, and 104 gains, while UPD disorders, i.e., copy number neutral ROH confined to a single chromosome or genome-wide UPD, were detected in 10 patients [chromosomes 7 (2×), 14 (2×), 15 (4×), and genome-wide UPD (2×)]. Both PCNV and UPD were detected in one patient. The ES reads for rare variants in the 2 patients with genome-wide UPD and their parents were consistent with mosaic paternal UPD. The findings of 190 PCNVs and 6 UPD in approximately half (N=183, 54.5%) of the cases with PCNV/UPD findings, were new and not known prior to ES (Fig. 1b). Thus, PCNV/UPD findings from the QC array contributed to additional diagnoses in 183/11,020 (1.7%) of the ES patients studied. The 159 PCNVs and 4 UPD in the remaining 153 case (45.5%) had been detected by clinical copy number testing prior to ES. For these patients, the reason for ES testing was often to further investigate a potential molecular explanation for the etiology of additional phenotypes not fully explained by the PCNV/UPD alone. The 203 deletions identified by the QC array are primarily heterozygous except for 15 homozygous and 14 hemizygous deletions. The size range of heterozygous deletions was 14 kb to 96 Mb (median 1.4 Mb), while the duplications were 0.2 to 106 Mb in size (median 2.9 Mb). The homozygous/hemizygous deletions ranged from only 113 bp to 1.6 Mb in size. There were 40 aneuploidy findings including monosomy X (*N* = 9, mosaicism in 5), trisomy 21 (*N* = 6), XYY (*N* = 8, mosaicism in 2), XXY (*N* = 7), XXX (*N* = 3), and XXYY (*N* = 1). In addition, acquired aneuploidy in somatic cells was revealed in 4 cancer patients including trisomy 8 in 2 patients, mosaic monosomy 7 in 1 patient, and trisomies 8, 9, and X in a female patient. Multiple PCNVs or UPD were present in 19 patients; 14 of whom had unbalanced rearrangements, 2 had aneuploidy in addition to pathogenic gains, 1 (WD8) had both UPD15 and a de novo gain in 10q, and 2 cancer patients had multiple somatic PCNVs.

The QC array detected isodisomy in 20 patients, 3 of whom were associated with UPD disorders while in the remaining 17 patients the involved chromosomes were not associated with imprinting disorders. These isodisomy findings included UPD2 (8×), UPD1 (4×), and UPD of chromosome 3, 4, 8, 9, or 22 (1× each). Eight causative homozygous pathogenic SNVs within the isodisomic chromosome, i.e., biallelic variation for a recessive disease trait when only one parent had a carrier allele, were identified by ES in 7 of the 20 isodisomic chromosomes. Notably, detection of mosaic maternal UPD1 facilitated interpretation of the finding of a mosaic pathogenic variant in the *ZMPSTE24* gene located in chromosome 1 in a patient [31].

### Detection of CNV/ROH by clinical CMA

A subset (*N* = 3229) of 11,020 ES cases also had clinical CMA, among whom 197 PCNVs and isodisomy 7 were detected in 184 patients (Fig. 1c). The PCNVs included 22 aneuploidies, 65 gains, and 110 losses (6 homozygous/4 hemizygous). The losses ranged from 268 bp to 96 Mb in size (median 1.2 Mb), while the gains ranged from 596 bp to 106 Mb in size (median 3.2 Mb). Mosaicism was observed in 13 patients including 7 patients with aneuploidy, 5 patients with a gain, and 1 patient with a loss. Fourteen patients had two PCNVs each. In addition, copy number changes of unknown clinical significance were also identified, but these findings are not presented here.

For the patients without PCNVs/UPD by CMA, the QC array detected PCNVs/UPD in 8 patients including 7 patients with PCNVs and 1 patient with UPD15 (Fig. 1d, Table 1). Combination of the results from both CMA and the QC array showed that PCNVs/UPD were detected in 192 patients, 189 of whom are unrelated. The PCNV/UPD detection rate in the ES cases with CMA was 5.9% (189/3226) (Table 1). Diagnostic SNVs (not including homozygous/hemizygous deletion) by ES were identified in 919 unrelated patients (not including 9 affected siblings), giving a 28.5% (919/3220) diagnostic rate. The PCNVs/UPD detected by either CMA or the QC array are 22 aneuploidies, 2 UPD, and 179 PCNVs including 66 gains and 113 losses. Twenty-nine losses and 1 gain were smaller than 50 kb, which counts for 16.8% (30/179) of the detected PCNVs. Among the 1089 patients with molecular diagnoses, 23 unrelated patients had diagnoses consisting of both SNVs and CNVs including 19 patients with multiple diagnoses and 4 patients with compound heterozygous PCNVs and SNVs.

**Table 1:** Contribution to molecular pathogenic variant detection rate: PCNVs/UPD detected by CMA and/or the QC array in ES cases

Categories	ES patients with CMA (*N* = 3229)	Total ES patients (*N* = 11,020)
Total PCNV/UPD	192	373*
PCNVs by CMA	183	183
UPD by CMA	1	1
PCNVs by QC array only	7	181
UPD by QC array only	1**	9
PCNV/UPD detection rate^#^	5.9% (189/3226)	3.3% (367/11,014)
SNV detection rate^##^	28.5% (919/3220)	28.5% (3145/11,020)
Contribution of PCNV/UPD to diagnoses in molecularly diagnosed cases^	17.4% (189/1089)	10.6% (367/3475)

*One patient (WD8) had both a 0.7 Mb gain in 10q24 and UPD15

**The diagnosis of UPD in patient WU5 was considered to be by QC array only

^#^Affected siblings in three families with PCNVs that were detected by CMA, affected siblings in three families with PCNVs detected by the QC array, and affected siblings in nine families with SNVs from ES were counted as one family

^##^Does not include cases with autosomal recessive gene deletions or other PCNVs. The SNV detection rate for total ES cases was assumed to be the same as in ES with CMA

^Multiple diagnoses consisting both PCNV/UPD and SNV were detected in 37 unrelated ES patients including 19 patients with CMA

For the remaining 7791 cases without CMA, PCNVs/UPD were detected by the QC array in 181 cases, of whom 179 are unrelated. For all the ES cases with or without CMA performed at BG, 367 patients had PCNVs/UPD from either CMA or the QC array and PCNVs/UPD contributed to the diagnoses in 10.6% (367/3475) of patients with a molecular diagnosis (Table 1).

**Fig. 2:**
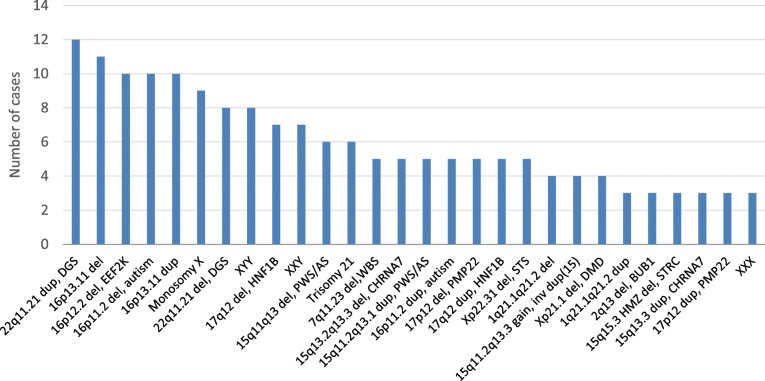
Most frequent PCNVs in 11,020 ES cases

The most frequent PCNVs detected in this ES cohort are shown in Fig. 2. The PCNVs observed in 10 or more patients are 22q11.21 duplication in the DiGeorge syndrome (DGS) region, 16p13.11 deletion and duplication, 16p12.2 deletion, and 16p11.2 deletion associated with autism. All of these CNVs have been associated with clinical variability and/or reduced penetrance. PCNVs from ES that are associated with unique syndromic features, such as DGS deletion, were underrepresented compared with the common findings in CMA. The frequency of the DGS deletion (*N* = 8), Williams-Beuren syndrome (WBS) deletion (*N* = 5), and 47,XXY (*N* = 7) in the ES patients with PCNVs is 5.3% (20/374), which is much lower than the reported frequency of 11.3% (57/506) in patients referred only for CMA [12]. Some of these abnormalities (5 DGS, 1 WBS, 5 XXY) were detected by CMA outside or at BG before ES while 6 of these abnormalities (2 DGS, 3 WBS, 1 XXY) were detected by concurrent CMA and ES.

### PCNV/UPD detection rate is higher when ES and CMA were performed concurrently

To better understand the contribution of CNVs to molecular diagnosis of ES, we divided the 3229 ES cases with CMA into three categories according to the timing of CMA testing. About 60% (1977/3229) of patients had CMA performed before ES (Table 2). The majority of these patients were negative for CMA although PCNVs and isodisomy 7 were indeed detected by prior CMA in 84 patients. Fourteen of these 84 patients had diagnostic SNVs identified by ES including 12 patients with two diagnoses consisting of both CNV and SNV. PCNVs/UPD were detected by CMA and/or the QC array in 4.6% (91/1977) cases, contributing to the molecular diagnoses in 12.5% of the molecularly diagnosed cases. We explored the reason why ES was ordered while the prior CMA was positive. The main reason is that the clinical phenotypes observed by the clinician cannot be completely explained by the CMA findings as seen in 63 out of 84 patients (Table 2). For example, phenotypes could not be fully explained by aneuploidy of sex chromosomes in 10 patients and trisomy 21 in 2 patients. For the remaining 21 patients, in 19 patients, the pathogenic CNVs were not obviously relevant to the clinical phenotypes; in 1 patient, a heterozygous deletion detected by CMA was considered as pathogenic after the subsequent detection of a disease-causing SNV, and in 1 patient, a homozygous exonic deletion was detected by targeted reanalysis of CMA data.

**Table 2:** Contribution of PCNV/UPD to molecular diagnoses when CMA was performed before, concurrently, or after ES

	Number of patients	PCNV/UPD by CMA	PCNV/UPD detection rate^#^	SNV detection rate*	Contribution of PCNV/UPD in molecularly diagnosed cases
ES + CMA	3229	184	5.9% (189/3226)	28.5% (919/3220)	17.4% (189/1089)
CMA done before ES	1977	84	4.6% (91/1977)	32.6% (643/1972)	12.5% (91/727)
Partially solved		63			
Inconclusive/non-diagnostic		19			
Positive only once combined with ES data		1			
Missed by CMA in original report		1			
Concurrent CMA/ES	1045	75	7.2% (75/1042)	24.1% (251/1041)	23.6% (75/318)
Solved without ES		49			
Partially solved		22			
Inconclusive/non-diagnostic		3			
Positive only once combined with ES data		1			
CMA done after ES	207	22	11.1% (23/207)	12.1% (25/207)	50.0% (23/46)
Solved		16			
Partially solved		5			
Positive only once combined with ES data		1			

^#^PCNV/UPD detection rate calculation includes the PCNVs/UPD detected by CMA and/or the QC array

*Does not include cases with autosomal recessive gene deletions or other PCNVs

About one third (1045/3229) of patients had CMA and ES performed concurrently. PCNVs/UPD were detected by CMA and/or the QC array in 7.2% (75/1042) cases, contributing to the diagnoses in 23.6% of molecularly diagnosed cases. The CMA findings explained the main phenotypes in the majority of patients with PCNVs (49/75), provided a partial diagnosis in 22 patients, while the contribution of CNVs to the phenotypes were inconclusive in 3 patients.

Only 6% (207/3229) of patients had CMA testing after ES results were obtained, of whom 23 patients had PCNVs. All of the PCNVs were previously detected by the QC array and/or ES read depth data, and CMA was applied as a confirmatory testing, except for a small < 4 kb deletion in *SLC7A7* [32].

While the diagnostic rate by SNVs for the 3229 ES cases with CMA was 28.5%, the detection rates were quite different among these categories (Table 2). ES cases with prior CMA had the highest molecular detection rate of 32.6%, while the lowest rate of 12.1% was seen for the cases with CMA done after ES. Concurrent ES/CMA had a SNV diagnostic rate of 24.1%, between the other two categories.

### Comparison of the CNV/ROH findings from CMA and the QC array

Of the 336 patients with findings from the QC array, CMA was not done for the 182 cases while the other 154 had CMA performed at BG (Fig. 1d). The findings from the QC array were also observed by CMA in 146 cases. Only 7 patients had a CNV by the QC array which was not detected by CMA. Among these patients, 2 had exonic single gene deletions that were not detectable in array v8.1.1 that does not have exonic coverage for the genes involved, 3 had deletions that were missed by early version BAC arrays, 1 had a somatic 39 Mb gain detected on a blood sample in a leukemia patient while CMA performed on tissues was negative, and 1 had a 51 Mb gain in an affected tissue detected by the QC array while CMA on a blood sample was negative. Of the 10 patients with findings related to UPD disorders by the QC array, 2 had CMA performed using an Agilent array with SNP probes. One patient (WU1) had isodisomy 7 detected by both QC array and CMA. The other patient (WU5) had an ~ 35 Mb ROH detected by the QC array and UPD15 was confirmed by subsequent methylation study. The large ROH was also observed in the reanalysis of the CMA data. For isodisomies in non-imprinting chromosomes that were detected by the QC array, only 2 cases had CMA with SNP probes and in both cases isodisomy 2 was also detected.

The PCNVs detected by CMA were also observed by the QC array for the majority of cases (79%, 145/183) but not in all 183 cases because of the lower resolution of the QC array (Fig. 1d). The PCNVs that were detected by CMA but not by the QC array were seen in 38 patients, including 14 gains and 25 losses; the gains were smaller than 750 kb, and the losses were smaller than 76 kb except for four gains of 1 to 3 Mb and one loss of 1.5 Mb.

### Dual molecular diagnoses consisting of both CNV and SNV

Multiple molecular diagnoses of differing variant types derived from ES and the QC array data were detected in 38 patients (Table 3). In 36 patients, the PCNV detected by the QC array and the SNV detected by ES were in different genomic regions. In 2 patients (WD6 and WD14), SNVs were located within the regions of the PCNVs. These 2 patients had point mutations in *SUMF1* and *NDE1*, within large deletions in 3p26.3p26.1 and 16p13.11, respectively. The SNVs were apparently homozygous in the sequencing data, but in conjunction with the PCNV findings, the SNVs were precisely interpreted as compound hemizygous changes.

**Table 3:** Patients (*N* = 38) with two or more diagnoses consisting of SNVs detected by ES and PCNVs detected by arrays

Patient ID	Findings from arrays				Findings from ES		
	CNVs	Size (Mb)	Associated disorders	Known prior to ES	SNVs	Associated disorders	Inheritance
WD1	1p36.33 del, 8p23.3p22 gain	0.715, 13.753	Unbalanced translocation	No	HMZ c.11754G>A (p.W3918*) pathogenic variant in *USH2A*	Usher syndrome 2A [MIM:276901]	AR
WD2	1q21.1q21.2 del, inherited	0.847	1q21.1 deletion syndrome [MIM:612474]	Yes	HTZ c.1396_1399dup (p.Y467fs) pathogenic variant in *ZFPM2*	Tetralogy of Fallot [MIM: 187500]; diaphragmatic hernia 3 [MIM:610187]	AD
WC2*^, #^,3*^, #^	1q21.1q21.2 del	1.207	1q21.1 deletion syndrome [MIM:612474]	No	HTZ c.283C>T (p.R95*) pathogenic variant in *CTNNB1,* dn	Mental retardation, autosomal dominant 19 [MIM:615075]	AD
WD3	1q24.2q25.3 gain	10.944	Large gain in 1q	Yes	HTZ c.182A>G (p.H61R) likely pathogenic variant in *PTEN*, dn	Cowden disease [MIM:158350]	AD
WD4	2q21.1 del	0.447	2q21.1 deletion including *ARHGEF4* and *GPR148* [PMID:22543972]	No	HTZ c.1615delG (p.E539fs) pathogenic variant in *PTCH1*	Basal cell nevus syndrome [MIM:109400]	AD
WD5	2q36.3 del	0.004	Early language delay and cerebral white matter hyperintensities [PMID:23810381]	Yes	HTZ c.2155+2T>C pathogenic variant in *CASK*	Mental retardation and microcephaly with pontine and cerebellar hypoplasia [MIM:300749]	X-linked
WD6	3pter del	5.15	Distal 3p deletion syndrome [MIM:613792]	No	HMZ c.836C>T (p.A279V) VUS in *SUMF1*	Multiple sulfatase deficiency [MIM:272200]	AR
WD7	3q21.2q21.3 del	2.053	Global developmental delay, absent or hypoplastic corpus callosum, and dysmorphic facies [MIM:617260]	No	HTZ c.706C>T (p.R236*) pathogenic variant in *ACAN*	Spondyloepiphyseal dysplasia type Kimberley [MIM:608361]	AD
WD20	4p16.1 gain, mosaic, dn	1.112	4p16.1 duplication [PMID: 15378535]	Yes	HMZ c.882_883del (p.Q295fs) pathogenic variant in *WDR45*, dn	Brain iron accumulation 5 [MIM:300894]	X-linked
WD8**	10q24.31q24.32 gain, dn	0.736	Split hand-split foot malformation 3 [MIM:246560]	No	HMZ c.2209G>C (p.G737R) pathogenic variant in *POLG*	Leigh syndrome [MIM:256000]	AD
WD9	15q13.2q13.3 del BP4-5	1.477	15q13.3 microdeletion syndrome [MIM:612001]	No	HTZ c.503G>A (p.R168H) pathogenic variant in *TPM3*, dn	Nemaline myopathy 1 [MIM:609284]; Cap myopathy 1 [MIM:609284]	AD
WD22	15q15.3 HMZ del	0.049	Deafness, autosomal recessive 16 [MIM:603720]	No	HMZ c.73G>T (p.E25*) pathogenic variant in *EPCAM*	Diarrhea 5, with tufting enteropathy, congenital [MIM:613217]	AR
WD13*	16p13.11 del, dn	1.166	16p13.11 microdeletion syndrome. [PMID:24105370]	Yes	HTZ c.5351T>C (p.V1784A) likely pathogenic variant in *SCN1A*, dn	Epileptic encephalopathy, early infantile, 6 [MIM:607208]	AD
WD23	16p13.11 del	1.166	16p13.11 microdeletion syndrome. [PMID:24105370]	Yes	HTZ c.2350_2351dupTG (p.W784fs) pathogenic variant in *NF1*	Neurofibromatosis-Noonan syndrome [MIM:601321]	AD
WD14	16p13.11 del, inherited	0.770	16p13.11 microdeletion syndrome. [PMID:24105370]	Yes	HMZ c.872C>T (p.S291F) VUS in *NDE1*	Lissencephaly type 4 [MIM:614019]	AR
WD15	16p13.11 gain	1.314	16p13.11 microduplication [PMID:21150890]	Yes	HMZ c.1156C>T (p.R386W) pathogenic variant in *FDXR*	Auditory neuropathy and optic atrophy [MIM:617717]	AR
WD15	16p13.11 gain	1.314	16p13.11 microduplication [PMID:21150890]	Yes	HTZ c.8045delT (p.L2682fs) pathogenic variant in *KMT2A*	Wiedemann-Steiner syndrome [MIM:605130]	AD
WD30	16p13.11 gain, inherited	1.165	16p13.11 microduplication [PMID:21150890]	Yes	HTZ c.1215delA (p.P405fs) pathogenic variant and c.1051A>C (p.T351P) VUS in *ROBO3*	Familial horizontal gaze palsy with progressive scoliosis [MIM:607313]	AR
WD21	16p13.11 gain, inherited	1.166	16p13.11 microduplication [PMID:21150890]	No	HTZ c.356T>C (p.L119P) pathogenic variant in *KRT13*, dn	White sponge nevus 2 [MIM:615785]	AD
WD12	16p12.2 del	0.387	16p12.2 microdeletion (*EEF2K*) [PMID:27848943]	Yes	HTZ c.6619_6621delGAG (p.E2207del) pathogenic variant in *SPTAN1*, dn	Epileptic encephalopathy, early infantile, 5 [MIM:613477]	AD
WD35	16p11.2 loss, inherited	0.377	16p11.2 deletion syndrome, 220kb [MIM:613444]	Yes	HTZ c.3280delG (p.E1094fs) in *KAT6B*, dn	Ohdo syndrome, SBBYS variant [MIM:603736]	AD
WD33	16p11.2 gain	0.521	16p11.2 duplication syndrome [MIM:614671]	Yes	HTZ c.1618dupC (p.Q540fs) pathogenic variant in *TCF20*, dn	Autism, intellectual disability, and postnatal overgrowth [PMID:27436265]	AD
WD10*	16p11.2 del, inherited	0.526	16p11.2 deletion syndrome, 593Kb [MIM:611913]	Yes	HTZ c.3505C>T (p.R1169*) likely pathogenic variant in *KAT6A*; inherited from similarly affected father	Mental retardation, autosomal dominant 32 [MIM:616268]	AD
WD11	16p11.2 del	0.526	16p11.2 deletion syndrome, 593Kb [MIM: 611913]	No	HTZ c.649dup (p.R217fs) pathogenic variant in *PRRT2*	Familial paroxysmal kinesigenic dyskinesia [MIM:602066]	AD
WD16	17p12 del, dn	1.448	HNPP [MIM:162500]	Yes	HTZ c.1264C>T (p.R422*) pathogenic variant in *DEPDC5*, dn	Epilepsy, familial focal, with variable foci [MIM:604364]	AD
WD24	17p12 del	1.294	HNPP [MIM:162500]	Yes	HTZ c.607G>A (p.G203R) likely pathogenic variant in *GNAO1*, dn	Epileptic encephalopathy, early infantile, 17 [MIM:615473]	AD
WD17*	17q11.2 gain	1.072	17q11.2 microduplication syndrome [MIM:613675]	No	HTZ c.1286G>A (p.W429*) pathogenic variant in *SOX11*	Mental retardation, autosomal dominant 27 [MIM:615866]	AD
WD25	17q12 del	1.776	Renal cysts and diabetes syndrome [MIM:137920]	No	Compound HTZ c.2099C>T (p.P700L) and c.2734C>T (p.R912W) VUS in *KIF1C*	Spastic ataxia 2, autosomal recessive [MIM:611302]	AR
WD28	17q12 gain, inherited	1.348	17q12 duplication [PMID:22241097]	Yes	HTZ c.1592G>T (p.W531L) likely pathogenic variant in *BRAF*, dn	Cardiofaciocutaneous syndrome 1 [MIM:115150]	AD
WD26	20p12.2p11.23 gain, inherited	7.614	Large gain in 20p	Yes	HTZ c.310C>G (p.R104G) VUS in *MKL2*, dn	Autism [PMID:22495311,23,375,656]	AD
WD18	22q11.21 gain, dn	2.568	22q11.2 microduplication syndrome [MIM:608363]	Yes	HMZ c.664delC (p.L222fs) pathogenic variant in *AP4B1*	Autosomal recessive spastic paraplegia 47 [MIM:614066]	AR
WD27	22q11.21q11.23 del	1.833	22q11.2 distal deletion [MIM:611867]	Yes	HTZ c.1574C>T (p.P525L) pathogenic variant in *FUS*	Amyotrophic lateral sclerosis 6, with or without frontotemporal dementia [MIM:608030]	AD
WD34	22q13.33 loss, dn	0.012	22q13.3 deletion syndrome [MIM:606232]	No	HTZ c.1474_1485del (p.Y492_F495del) likely pathogenic variant in *FOXP1*, dn	Mental retardation with language impairment and autistic features [MIM:613670]	AD
WD19*	Xp22.31 del	1.413	X-linked ichthyosis in male [MIM:308100]	Yes	HMZ c.1376_1377del (p.C459*) pathogenic variant in *KIAA2022*, dn	Mental retardation, X-linked 98 [MIM:300912]	X-linked
WD31	Xp21.1 HMZ del	0.001	Becker muscular dystrophy [MIM:300376]	Yes	HTZ c.709G>A (p.E237K) pathogenic variant in *KIF5C*, dn	Complex cortical dysplasia with other brain malformations 2 [MIM:615282]	AD
WC17	Xq28 del	0.228	Mental retardation, X-linked, FRAXE type [MIM:309548]	No	HTZ c.3136-2A>G pathogenic variant in *ARID1B*	Coffin-Siris syndrome 1 [MIM:135900]	AD
WD29	47,XXY	Entire chrX	47,XXY	Yes	HTZ c.1726C>T (p.R576W) pathogenic variant (dn) and c.1108-5G>A VUS in *ATAD3A*	Harel-Yoon syndrome [MIM:617183]	AR, AD
WD32	47,XYY	Entire chrY	47,XYY	Yes	HTZ c.1478dupT (p.S494fs) pathogenic variant in *DYRK1A*, dn	Mental retardation, autosomal dominant 7 [MIM:614104]	AD

*HNPP* neuropathy, recurrent, with pressure palsies, *AD* autosomal dominant, *AR* autosomal recessive, *VUS* variant of unknown significance, *dn* de novo, *del* deletion, *HTZ* heterozygous, *HMZ* homozygous or hemizygous

*Reported previously [35]

**WD8 also had UPD15

^#^WC2 and WC3 are siblings

Two of these patients had three molecular diagnoses (Table 3); patient WD8 had UPD15 in addition to the pathogenic CNV and SNV in different loci, while patient WD15 had a 16p13.11 gain, a heterozygous pathogenic variant in *KMT2A* and a homozygous pathogenic variant in *FDXR*. In 16 patients, recurrent aberrations known to be associated with incomplete penetrance were identified including 1q21 deletion in 3 patients (2 were twins), 16p13.11 deletion in 3 patients and duplication in 3 patients, 16p11.2 deletion associated with autism in 2 patients and gain in 1 patient, and 2q21.1 deletion, 16p12.2 deletion, 17q11.2 gain, 22q11.21 gain, each in 1 patient. PCNVs in 23 patients were known prior to ES, 8 of which are known to be associated with incomplete penetrance.

Pathogenic SNVs were detected in 2 cancer patients, 1 with 1q gain/7q loss, and the other with trisomy 8. Since these CNV changes are interpreted as somatic changes, these 2 were not included in the list of dual diagnoses.

### CNV detection led to the diagnoses of recessive disorders

**Fig. 3:**
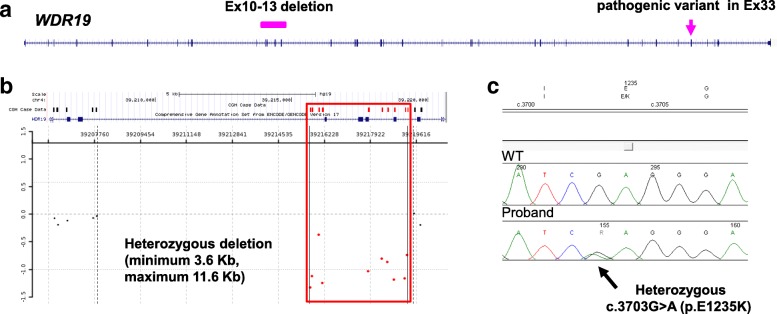
Detection of a gross deletion and a heterozygous pathogenic variant in the *WDR19* gene (RefSeq NM_025132) in patient WC5 with a history of end-stage renal disease. **a** The locations of the deletion and single nucleotide variant in *WDR19*. The exons 10–13 deletion is indicated by a bar, and the pathogenic variant is indicated by an arrow. **b** A plot to show the deletion detected by CMA. The deletion is indicated by the red box, and the probes are indicated by bars on the top. **c** Chromatograph trace to show the heterozygous pathogenic variant detected by ES and confirmed by Sanger sequencing

Compound heterozygous CNV and SNV affecting single disease genes were detected in 4 patients. Two of them had a SNV outside the CNV regions, while the other 2 had a SNV which is inside the deleted region. These CNVs were detected by CMA with exonic coverage of disease genes and not detectable by the QC array. Both ES and CMA findings were required for the diagnoses of patients WC5 and WC27 who had a combination of a heterozygous SNV and a heterozygous deletion or duplication in an AR gene. In WC5, ES detected a heterozygous c.3703G>A (p.E1235K) pathogenic variant in exon 33 of the *WDR19* gene and CMA detected a ~ 3.6 kb deletion of exons 10–13 of the *WDR19* gene (Fig. 3). Parental studies were not performed, and therefore, the phase of these changes was unknown. Patient WC27 had severe combined immunodeficiency, a pathogenic variant in one allele of the *ADA* gene that was detected by ES, and a gain of 596 bp including exon 2 of *ADA* in the other allele that was detected by CMA [33]. The other 2 patients with a SNV inside the deleted region have been described before [32, 34, 35]. An apparently homozygous pathogenic SNV in the disease genes *SLC7A7* or *CRIPT* was detected in the probands by ES; however, only one parent had the heterozygous variant and the other parent was negative. Subsequent CMA or targeted analysis of the previous CMA data detected the suspected deletion encompassing the SNV.

In addition to compound heterozygous CNV and SNV, homozygous deletions in autosomes were detected in 17 patients (Table 4). The recurrent homozygous 15q15.3 *STRC* gene deletion was detected in 3 patients referred for hearing loss, and a homozygous 16p13.3 *HBA1/HBA2* deletion was detected in 2 additional patients. Interestingly, the QC array detected a homozygous 2.2 kb deletion affecting exon 6 of the *WWOX* gene, which is flanked by heterozygous deletions in patient WH12. The QC array also showed that each of the parents carried a different but overlapping heterozygous deletion. Therefore, the patient actually had compound heterozygous deletions in *WWOX* (Additional file 1: Figure S2), a situation similar to that observed in the *ATAD3A* disease gene discovery [28].

**Table 4:** Homozygous pathogenic deletions (*N* = 17) detected by the QC array and/or CMA

Patient ID	Genomic intervals (hg19)	Chromosome bands	Size (Kb)	Disease genes	Exons deleted	Total exons	RefSeq^#^
WH1	chr2:31758677-31805700	2p23.1	47.023	*SRD5A2*	3	6	NM_000348.3
WH2*	chr2:110862477-110983703	2q13	121.226	*NPHP1*	1 to 19	19	NM_000272.2
WH3	chr2:169824976-169830328	2q31.1	5.352	*ABCB11*	13 to 17	28	NM_003742.2
WH4*	chr5:140953993-140992629	5q31.3	38.636	*DIAPH1*	2 to 15	28	NM_005219.3
WH5,6, WD22	chr15:43890409-43939659	15q15.3	49.25	*STRC*	1 to 29	29	NM_153700.2
WH8*,9	chr16:221962-228406	16p13.3	6.444	*HBA1; HBA2*	1 to 3; 1 to 3	3; 3	NM_000558.3;NM_000517.3
WH10	chr16:1557663-1561126	16p13.3	3.463	*IFT140*	31	31	NM_014714.3
WH11*	chr16:78143268-78154701	16q23.1	11.433	*WWOX*	3 to 4	9	NM_016373.1
WH12^#^	chr16:78409180-78431277	16q23.1-q23.2	22.097	*WWOX*	6	9	NM_016373.1
WH13	chr16: 78458774-78463512	16q23.1	4.738	*WWOX*	7	9	NM_016373.1
WH14	chr17:9489128-9489263	17p13.1	0.135	*CFAP52*	2	14	NM_145054.4
WC14*	chr19:55652193-55665240	19q13.42	13.047	*TNNT1; TNNI3*	1 to 9; 8	14; 8	NM_003283; NM_000363
WH15*	chr3:190039387-190040504	3q28	1.117	*CLDN1*	1	4	NM_021101.4
WH16*	chr16:15758011-15761384	16p13.11	3.373	*NDE1*	3 to 4	10	NM_001143979.1

*The deletions were detected by CMA performed at BG. All these 17 deletions were detected by the QC array except for WH15 and WH16. The genomic intervals were based on CMA data when available

^#^WH12 had compound heterozygous overlapping deletions

### UPD disorders in ES cases

UPD disorders were detected in 10 patients through detection of large ROH by the QC array (Table 5). The ROH findings included two UPD7 (entire chromosome), two UPD14 (39 Mb, *N* = 1; 33 Mb, *N* = 1), four UPD15 (35 Mb, *N* = 1; 32 Mb and 6 Mb, *N* = 1; entire chromosome, *N* = 1; 17 Mb, *N* = 1), and two mosaic genome-wide paternal UPD. Three patients had isodisomy, while the other patients had heterodisomy with segmental isodisomy. The origin of the UPD chromosomes was determined by the inheritance of rare variants detected by ES or by additional methylation studies. The two isodisomy 7, one maternal UPD15 and one genome-wide UPD, were known prior to ES.

**Table 5:** Large ROH detected by arrays were shown to be associated with UPD disorders in 10 patients

Patient ID	Chromosome	ROH	Confirmation study	Type of UPD	Molecular Diagnosis	Clinical findings
WU1*^,#^,2^#^	7	Entire chr7	Parental studies of rare variants	Isodisomy	Maternal UPD7	Silver-Russell syndrome
WU3	14	39 Mb	Parental studies of rare variants	Heterodisomy	Maternal UPD14	Maternal UPD14 syndrome
WU4	14	33 Mb	Parental studies of rare variants	Heterodisomy	Maternal UPD14	Maternal UPD14 syndrome
WU5*	15	35 Mb	Methylation studies	Heterodisomy	Paternal UPD15	Angelman syndrome
WU6	15	32 Mb + 6 Mb	Parental studies of rare variants	Heterodisomy	Maternal UPD15	Prader-Willi syndrome
WU7	15	Entire chr15	Parental studies of rare variants	Isodisomy	Paternal UPD15	Angelman syndrome
WD8^#^	15	17 Mb	Known before ES	Heterodisomy	Maternal UPD15	Prader-Willi syndrome
WU8,9^#^	1–22	Genome-wide	None	Isodisomy	Paternal	Mosaic genome-wide UPD

^#^UPD in patients WU1, 2, 9 and WD8 were known before ES testing

*Isodisomy 7 in WU1 and ROH in WU5 were also detected by CMA at BG

### Association of PCNV/UPD detection rate and phenotypic profile of patients

**Fig. 4:**
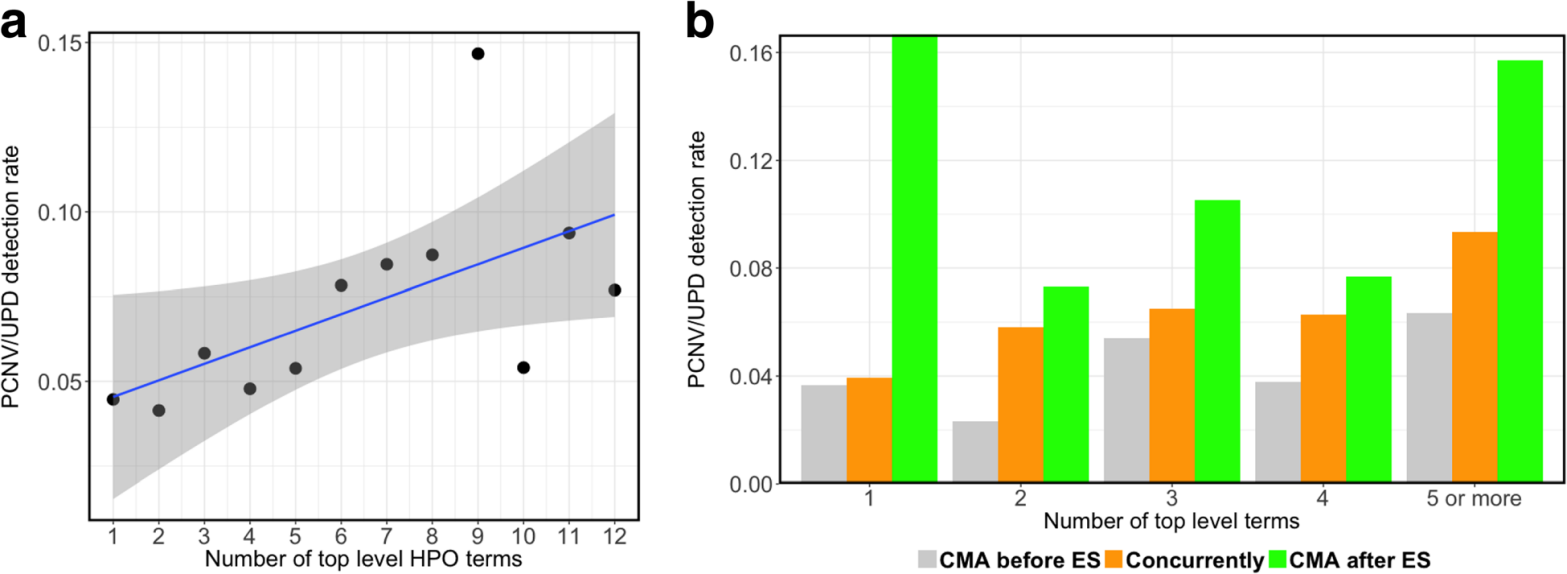
PCNV/UPD detection rate increases with an increase in the number of distinct top-level HPO terms. **a** A scatterplot showing the trend of PCNV/UPD detection rate as a function of the number of top-level terms. The line represents the fit of a linear model with the shaded area corresponding to the 95% confidence interval. **b** A bar chart to show the PCNV/UPD detection rate vs number of top-level term for each category of ES divided based on the timing of CMA

As the PCNV/UPD detection rate of 5.9% in ES patients with CMA is much lower than the previously reported 15–20% diagnostic yield by CMA alone, we explored the possibility of ascertainment bias in our cohort due to variables such as patients’ clinical features, age, and sex. We annotated a subset (*N* = 2876) of patients’ clinical features using HPO terms and mapped them to the corresponding top-level HPO terms. We stratified these patients into sub-cohorts each of which shared a different number of unique top-level HPO terms. We found a significant effect of the number of top-level HPO terms in a sub-cohort on the PCNV detection rate in these cohorts. The PCNV detection rate ranged from 4.1 to 14.6% (overall 6%) depending on the number of unique top-level HPO terms in a cohort (Fig. 4a). There was an increase in the PCNV detection rate with an increase in the number of distinct top-level HPO terms for ES patients with CMA at BG; this trend was apparent especially for concurrent ES and CMA (Fig. 4b). These results are consistent with CNV-positive rate increasing with the syndromic nature of the clinical presentation.

We also explored whether certain specific clinical features were more likely to be associated with a molecular diagnosis by CNVs by comparing the PCNV detection rate among patients with different phenotypes. PCNV detection rate significantly increased in cohorts that had abnormalities affecting the cardiovascular system compared to the group that did not include cardiovascular abnormality. Similar results were obtained for cohorts with abnormality in growth, the respiratory system, and the head or neck. However, patients with or without abnormalities in the nervous system had comparable PCNV/UPD detection rate (Table 6).

**Table 6:** The PCNV/UPD detection rate among ES patients having CMA at BG with or without a top-level HPO term

HPO term	HPO ID	PCNV/UPD detection rate with HPO term	PCNV/UPD detection rate without HPO term	Odds ratio	Fisher exact test (*p* value)
Abnormality of the cardiovascular system	HP:0001626	61/616 (9.01)	112/2087 (5.09)	1.84	0.0004
Abnormality of the respiratory system	HP:0002086	39/365 (9.65)	134/2338 (5.42)	1.86	0.0021
Growth abnormality	HP:0001507	70/805 (8)	103/1898 (5.15)	1.6	0.0037
Abnormality of the head or neck	HP:0000152	95/1210 (7.28)	75/1496 (4.77)	1.57	0.0053
Abnormality of connective tissue	HP:0003549	19/219 (7.98)	154/2484 (5.84)	1.4	0.1983
Abnormality of the immune system	HP:0002715	26/324 (7.43)	147/2379 (5.82)	1.3	0.231
Abnormality of blood and blood-forming tissues	HP:0001871	23/272 (7.8)	173/2703 (6.02)	1.32	0.2516
Abnormality of the ear	HP:0000598	25/312 (7.42)	148/2391 (5.83)	1.29	0.2712
Abnormality of the skeletal system	HP:0000924	84/1127 (6.94)	173/2703 (6.02)	1.16	0.2898
Abnormality of metabolism/homeostasis	HP:0001939	27/347 (7.22)	146/2356 (5.84)	1.26	0.294
Abnormality of the breast	HP:0000769	2/17 (10.53)	171/2686 (5.99)	1.85	0.3183
Abnormality of prenatal development or birth	HP:0001197	15/297 (4.81)	158/2406 (6.16)	0.77	0.3798
Abnormality of abdomen morphology	HP:0001438	39/543 (6.7)	134/2160 (5.84)	1.16	0.4354
Abnormality of the limbs	HP:0040064	24/322 (6.94)	149/2381 (5.89)	1.19	0.4687
Abnormality of the genitourinary system	HP:0000119	27/371 (6.78)	146/2332 (5.89)	1.16	0.4953
Abnormality of the eye	HP:0000478	40/576 (6.49)	133/2127 (5.88)	1.11	0.5669
Neoplasm	HP:0002664	3/72 (4)	170/2631 (6.07)	0.64	0.6242
Abnormality of the endocrine system	HP:0000818	9/177 (4.84)	164/2526 (6.1)	0.78	0.6314
Abnormality of the musculature	HP:0003011	62/989 (5.9)	111/1714 (6.08)	0.97	0.8709
Abnormality of the integument	HP:0001574	25/405 (5.81)	148/2298 (6.05)	0.96	0.9128
Abnormality of the nervous system	HP:0000707	139/2166 (6.03)	34/537 (5.95)	1.01	1

The vast majority of patients referred for ES were pediatric; only 6.6% (213/3229) of the ES patients with CMA at BG were adults at the time of ES testing. We did not detect any significant difference in ages in the CNV-positive and CNV-negative cohorts (Mann-Whitney test: *p* = 0.5536). Additionally, we did not detect any significant effect of age or sex contributing to the PCNV detection rate in a sub-cohort of 2876 individuals with HPO terms annotated (see the “Methods” section). The coefficients obtained from the logistic regression for the variables (number of top-level HPO term, age, and sex) were 0.11, 0.17, and − 0.01 respectively with only the number of top-level HPO terms being statistically significant (*p* < 0.001).

## Discussion

Our data demonstrate that CNV/ROH detection by microarrays in ES patients results in a higher diagnostic rate, leading to a more accurate and complete diagnosis. In this cohort of 11,020 ES patients, PCNVs/UPD were detected in 367 patients (3.3%), contributing to the molecular diagnoses in 10.6% of the molecularly diagnosed cased (Table 1).

Our data also demonstrates that CNV identification in ES patients uncovers instances of dual or more molecular diagnoses. Multiple molecular diagnoses associated with blended phenotypes can be a clinical diagnostic conundrum and are being increasingly found with the application of genome-wide technologies [35]. Both PCNVs and SNVs contribute to multiple diagnoses either through multilocus pathogenic variations or through additional recessive conditions caused by a pathogenic deletion in one allele and a SNV in the other allele. As we show in this study, without CNV detection, the 38 patients with multiple diagnoses would most likely obtain a partial diagnosis. From another perspective, the additional diagnoses from SNVs may be missed if ES is not pursued after a PCNV is identified in such patients.

CNV detection also ascertains diagnoses in AR disorders for which an etiological SNV was identified in one allele only (e.g., SNV of one exon and CNV involving other exons of the same gene) [29, 36], as shown in patient WC5 who had SNV and PCNV involving different exons of the *WDR19* gene. In addition, identification of PCNVs also facilitates accurate interpretation of the molecular genetics and transmission of genetics findings related to recessive disorders (e.g., deletion CNV + SNV at a locus versus homozygous SNV), allowing more accurate disease management and recurrence risk counseling. As seen in patients WD6 and WD14, without copy number analysis, a hemizygous SNV in one allele and a deletion in the other allele may be misinterpreted as a homozygous SNV instead of compound hemizygous SNV and heterozygous gross deletion.

Detection of large ROH may contribute to molecular diagnoses in ES. In this study, a diagnosis of UPD disorders was made in 10 patients through detection of large ROH confined to single chromosomes. In addition, SNP data from the microarray might provide supportive evidence of a deletion when the small deletion is not apparent in the QC array analysis as exemplified in the discovery of compound deletion and SNV in the *SLC7A7* gene [32]. Moreover, isodisomy may unmask a pathogenic SNV as seen in the 7 patients in this study who had homozygous SNVs in the chromosomes with isodisomy.

The majority of patients referred for ES or CMA are pediatric patients with primarily neurodevelopmental phenotypes. Our HPO term analysis showed similar PCNV/UPD detection rate for patients with or without abnormalities in the nervous system. However, the analysis revealed that PCNV detection rate increases with the increasing number of top-level HPO terms in the patients, suggesting that syndromic phenotypes are more likely to have a PCNV than an isolated phenotype. The HPO term analysis also revealed a significant higher PCNV detection rate for the phenotypes that map to “Abnormality of cardiovascular system” (Table 6). Consistently, high diagnostic yield has been previously reported for microarray testing of patients with congenital heart diseases [37]. In contrast to the relatively high yield from CMA, clinical ES for infants in NICU had a relatively low yield for cardiovascular abnormalities [17].

The PCNV/UPD detection rate (overall 3.3%, 5.9% for ES with CMA) is apparently lower than the 15–20% diagnostic yield in an earlier meta-analysis and ~ 10% from more recent studies [6, 10, 12]. Several lines of evidence indicate that the PCNV incidence in the ES patients does not reflect the PCNV incidence in CMA patient population. First, patients referred to ES often have had a long-term search for a genetic diagnosis and remained without a diagnosis after a series of genetic testing including microarray analysis [15]. As revealed in this cohort, CMA was done before ES in 60% of ES patients with CMA performed at BG and most of the ES patients without CMA at BG had prior CMA performed outside BG. The patients were referred to ES often after a negative CMA or occasionally when clinical features in the patients cannot be explained by the CMA findings. Second, the detection rate was higher (7.2% vs 4.6%) when CMA was ordered concurrently with ES compared to cases where CMA was performed prior to ES. This also indicates that the patients with pathogenic findings from prior CMA are less likely to be referred to ES. Third, comparing to CMA, a lower frequency of PCNVs associated with a high penetrance syndrome with typical features and less clinical heterogeneity, was observed in this study, which indicates such patients are less likely to be referred to ES. The additional factor contributing to the relatively lower detection rate is related to difference of CNV classification in this study compared with other studies. For example, 15q11.2 BP1-2 duplication was classified a change of unknown significance; however, this duplication is the most frequent PCNV in a clinical CMA study [12].

What could be the best approach to detect effectively and cost efficiently both SNVs and CNVs in a diagnostic laboratory? The concurrent QC array has lower PCNV detection sensitivity; however, the contribution from the QC array surpasses this limitation, especially considering the QC array is much cheaper than CMA. Alternative approaches include concurrent low coverage (or low-pass) whole-genome sequencing (WGS) or innovation in sequencing platform. Low-coverage whole-genome sequencing (0.25×) can cost efficiently detect > 50 kb CNVs within a short turn-around time [38, 39]. In addition, a combined sequencing platform comprising of focused exome and whole-genome backbone has been developed, which has the potential to detect deletions as small as 23 kb [40]. In general, only large CNVs can be reliably detected by the abovementioned approaches and smaller, especially exonic changes, may be undetected. In this study, 16.8% of CNVs that are < 50 kb would be possibly missed by the above approaches.

Currently, clinical CMA using arrays with exonic coverage for disease genes provide the most effective PCNV detection. Detection of exonic deletions or duplications can further significantly improve diagnosis of ES cases since up to 40% of intragenic CNVs can involve just one or two exons within a gene [25]. However, exonic duplications can be particularly challenging to detect [41]. As the first-tier diagnostic test for CNV detection, microarray with probe coverage for all exons within the targeted disease genes may circumvent this challenge [30]. ES and CMA can be done sequentially or concurrently. Performing ES only after a negative CMA testing is cost saving. However, it should be noted that ES is necessary when the CNVs cannot fully explain the patient’s clinical phenotype or are associated with reduced/uncertain penetrance, as shown by the finding of 38 dual diagnoses consisting of both CNVs and SNVs in this study, in a previous study on three families with intellectual disability and genomic imbalances [42] and in a case report of atypical Prader-Willi syndrome [43].

Concurrent CMA and ES provide simultaneous detection of point mutation changes, small indels, and large CNVs. This approach has enabled these tests to provide a molecular diagnosis at a detection rate of 7.2% for PCNVs/UPD and 24.1% for SNVs with CNV/ROH contributing to the diagnoses in 23.6% of the molecularly diagnosed cases (Table 2). Previous studies also showed that ES with simultaneous CMA yielded a higher diagnostic rate in autism spectrum disorders [44]. This concurrent approach is especially required in time-sensitive situations such as patients in the NICU. Moreover, this approach maximizes the recognition of multiple molecular diagnoses, i.e., pathogenic variation at more than one locus, from CNVs and SNVs since blended phenotypes resulting from dual diagnoses might not be noticeable without a molecular diagnosis [35, 43, 45]. In addition, cross-checking the data from both genome-wide assays allows for precise molecular diagnosis and better clinical care of AR diseases involving both CNVs and SNVs.

Performing CMA and ES sequentially or concurrently optimizes the molecular diagnoses of both CNVs and SNVs. However, the combined use of the two diagnostic methods in the clinical setting is labor intensive and time consuming in addition to the higher costs incurred. With the rapid advances of next-generation sequencing technologies, it is anticipated that WGS data may provide rare SNVs, insertion/deletions (indels < 50 bp), small CNVs, and genomic structural abnormalities. WGS is capable to accurately detect CNVs and also provide position and orientation information [46], having the potential to be the single test to detect pathogenic variants of all types (SNVs, indels, CNVs) and other genomic aberrations such as chromosomal anomalies (including aneuploidies and UPD).

## Conclusions

This study of a large cohort of 11,020 ES cases shows that copy number analysis increased molecular diagnostic rate, enabled dual molecular diagnoses, and provided more precise interpretation of genetic findings. In the 1045 ES cases with concurrent CMA, PCNVs/UPD contributed to the diagnoses in 23.6% of the molecularly diagnosed cases. While sequential or concurrent ES and CMA testing currently enables accurate diagnosis, further improvement of the next-generation sequencing platforms and data analysis pipelines and reanalysis of existing clinical genomics data will eventually improve molecular diagnostic rates; ultimately, a move towards clinical whole-genome sequencing may lead to effective CNV/SNV detection in a single test.

## Additional files

Additional file 1: **Figure S1.** An example to show that the QC array is highly reliable for ROH detection. Figure S2 Compound heterozygous deletions detected in patient WH12. (PPT 611 kb)

Additional file 2: Sensitivity of CNV detection for the QC array (DOCX 40 kb)

Additional file 3: Supplementary **Table S1.** Correlation of PCNV findings from CMA and the QC array in 496 ES cases (DOCX 44 kb)

## Abbreviations

ACMG: American College of Medical Genetics and Genomics
AOH: Absence of heterozygosity
AR: Autosomal recessive
BG: Baylor Genetics
CMA: Chromosomal microarray analysis
CNV: Copy number variant
DGS: DiGeorge syndrome
ES: Exome sequencing
HMZ: Homozygous or hemizygous
HPO: Human phenotype ontology
HTZ: Heterozygous
IBD: Identity-by-descent
NICU: Neonatal intensive care unit
PCNV: Pathogenic/likely pathogenic CNV
QC: Quality control
ROH: Runs of homozygosity
SNV: Single nucleotide variant
UPD: Uniparental disomy
VUS: Variant of unknown significance
WBS: Williams-Beuren syndrome
WES: Whole-exome sequencing
WGS: Whole-genome sequencing
