# Supplementary material for: Copy number variant and runs of homozygosity detection by microarrays enabled more precise molecular diagnoses in 11,020 clinical exome cases
Source: Genome Med. 2019 May 17;11:30. doi: 10.1186/s13073-019-0639-5 (PMC6525387; doi:10.1186/s13073-019-0639-5)
Supplement: Supplementary file 1 — Figure S1. An example to show that the QC array is highly reliable for ROH detection. Figure S2 Compound heterozygous deletions detected in patient WH12. (PPT 611 kb) [file 13073_2019_639_MOESM1_ESM.ppt]

## Slide 1
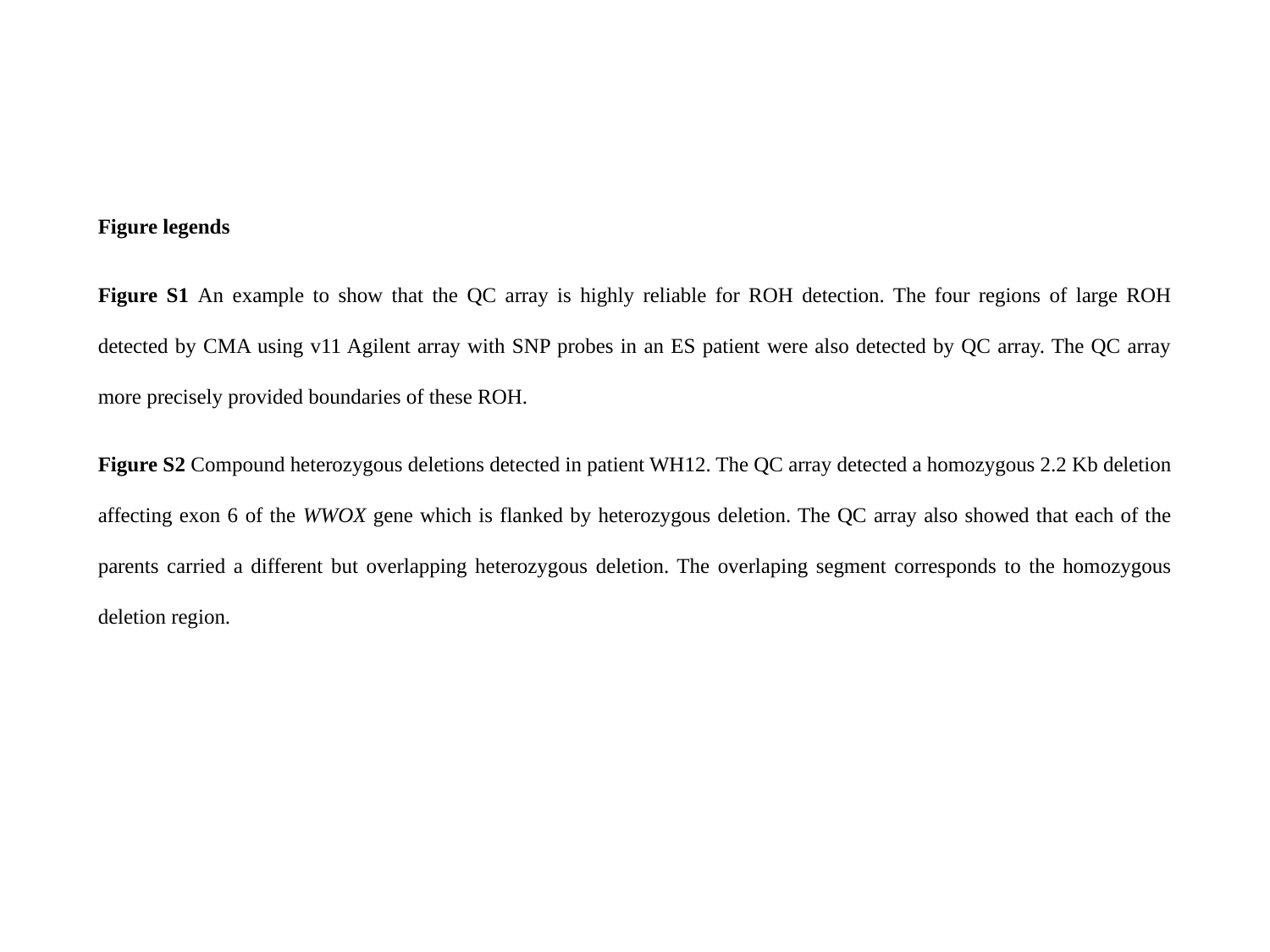

Figure legends
Figure S1 An example to show that the QC array is highly reliable for ROH detection. The four regions of large ROH detected by CMA using v11 Agilent array with SNP probes in an ES patient were also detected by QC array. The QC array more precisely provided boundaries of these ROH.
Figure S2 Compound heterozygous deletions detected in patient WH12. The QC array detected a homozygous 2.2 Kb deletion affecting exon 6 of the WWOX gene which is flanked by heterozygous deletion. The QC array also showed that each of the parents carried a different but overlapping heterozygous deletion. The overlaping segment corresponds to the homozygous deletion region.

## Slide 2
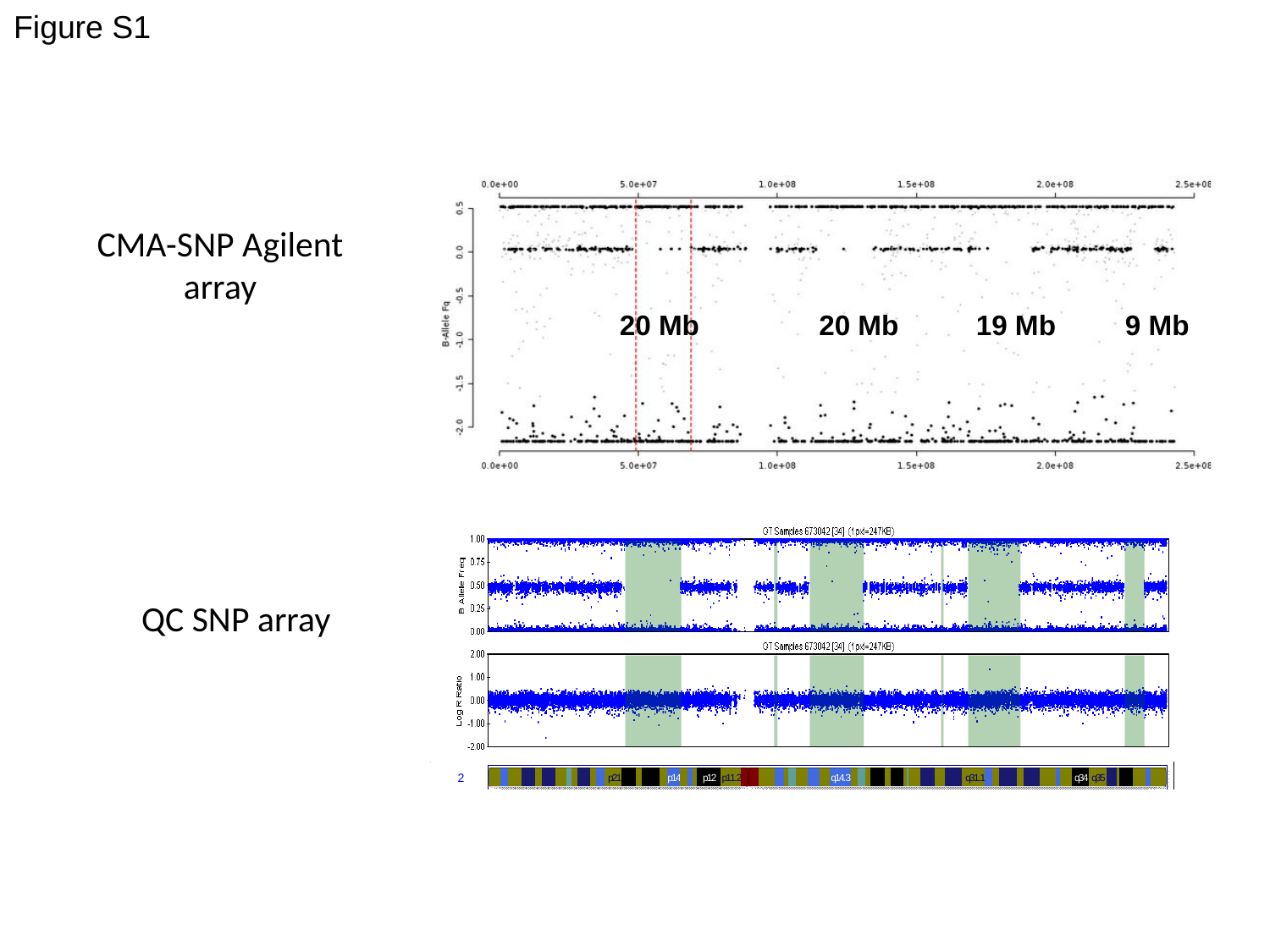

Figure S1
CMA-SNP Agilent array
20 Mb
20 Mb
19 Mb
9 Mb
QC SNP array

## Slide 3
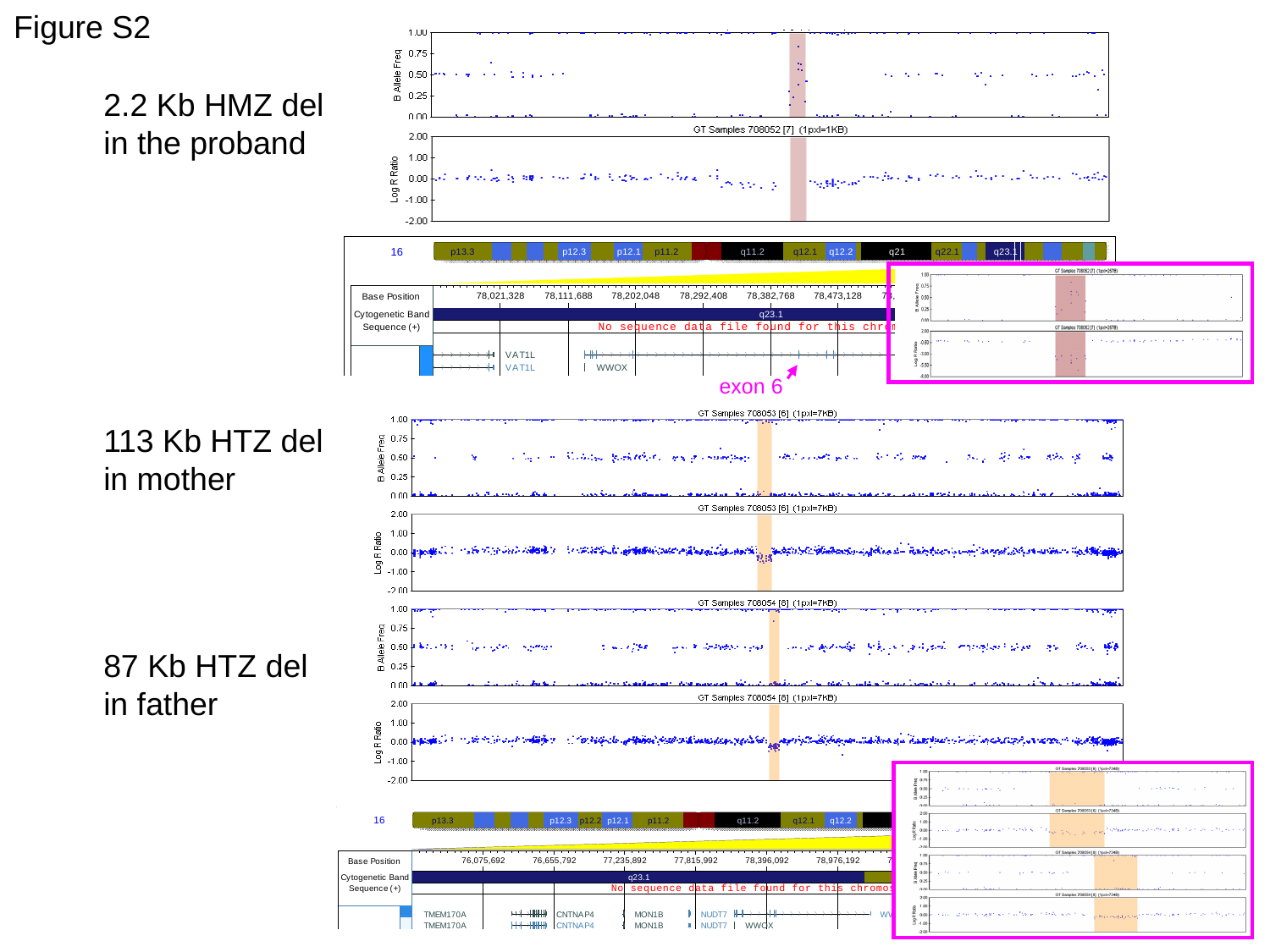

Figure S2
2.2 Kb HMZ del
in the proband
exon 6
Ex6
113 Kb HTZ del
in mother
87 Kb HTZ del
in father
