# Supplementary material for: Copy number variant and runs of homozygosity detection by microarrays enabled more precise molecular diagnoses in 11,020 clinical exome cases
Source: Genome Med. 2019 May 17;11:30. doi: 10.1186/s13073-019-0639-5 (PMC6525387; doi:10.1186/s13073-019-0639-5)
Supplement: Supplementary file 2 — Sensitivity of CNV detection for the QC array (DOCX 40 kb) [file 13073_2019_639_MOESM2_ESM.docx]

**Sensitivity of CNV detection for the QC array**

The QC array was performed for each ES case as a DNA sample and sequence quality control measurement. The QC array is known for its ability to detect copy number changes and copy number neutral ROH. However, the genotyping array used in conjunction with ES was not designed for clinical molecular diagnosis of copy number changes. To characterize the capability and limitation of the QC array in detection of clinically significant copy number changes, we compared the findings from the QC array and CMA in 496 cases with a concurrent ES and CMA testing that were ordered using as a combined test.

PCNVs detected by CMA included 18 losses and 9 gains from 25 unrelated patients as listed in Supplementary Table 1. Multiple CNVs were found in patients WC24 and WC25. Aneuploidy was detected in only one patient who had monosomy X. For the remaining patients with a single CNV, losses range from 1.7 Kb to 9.4 Mb and gains range from 596 bp to 3.0 Mb in size. PCNVs were detected in two pairs of siblings; an Xq28 gain including the *MECP2* gene was detected in two brothers and a 1q21.1q21.2 deletion was detected in two siblings from another family.

The QC array analyses detected 19/27 (70%) of the PCNVs that were identifiable by CMA. For the 18 pathogenic copy number losses detected by CMA, 15 were also detected by the QC array. These losses ranged in size from 0.2 to 9.5 Mb, to the whole chromosome X. Most of the deletions were larger than 1 Mb, while four deletions detected by the QC array were smaller than 0.6 Mb, including two ~ 0.5 Mb deletions in 16p12.2 and 1q21.3, respectively, a 0.2 Mb Xq28 deletion and a ~13 Kb homozygous 19q13.42 deletion of both *TNNT1* and *TNNI3* genes. Two deletions, which were smaller than 5 Kb and involving only single genes, *STX16* and *WDR19*, were detected by CMA but not detectable by the QC array. These were a deletion of 1.7 Kb in 20q13.32, including exons 6-7 of the *STX16* gene and a deletion of 4 Kb in 4p14 encompassing the exons 10-13 *WDR19* gene. Our data suggested that the QC array was able to detect most of the non-exonic pathogenic deletions, but may miss smaller sized deletion PCNVs, particularly for variants <10 Kb in size.

The sensitivity of detection for copy number gains is much lower than for copy number losses for the QC array. Only three of the nine pathogenic gains detected by CMA were also detected by the QC array including a 3.0 Mb gain in 22q11.1q11.21, a 2.5 Mb gain in 22q11.21 and a 2.4 Mb gain in 11q13.1q13.2. The gains not detected by the QC array ranged from 596 bp to 2.1 Mb, including a 2.1 Mb gain in 14q32.2q32.31, a 1.7 Mb gain in 16p13.11, and three gains smaller than 0.5 Mb.
