# Supplementary material for: Copy number variant and runs of homozygosity detection by microarrays enabled more precise molecular diagnoses in 11,020 clinical exome cases
Source: Genome Med. 2019 May 17;11:30. doi: 10.1186/s13073-019-0639-5 (PMC6525387; doi:10.1186/s13073-019-0639-5)
Supplement: Supplementary file 3 — Supplementary Table S1. Correlation of PCNV findings from CMA and the QC array in 496 ES cases (DOCX 44 kb) [file 13073_2019_639_MOESM3_ESM.docx]

**Supplementary Table 1** **Correlation of PCNV findings from CMA and the QC array in 496 ES cases**

| **Patient ID** | **Genomic region** | **CNV** | **Size**  **(Mb)** | **Associated genes and phenotype** | **Genes involved** | **Detected by**  **QC array** |
| --- | --- | --- | --- | --- | --- | --- |
| WC1 | 1p36.33p36.32 | loss | 1.99 | 1p36 deletion syndrome  [MIM:607872] | Multiple genes | yes |
| WC2,3 | 1q21.1q21.2 | loss | 1.207 | 1q21.1 deletion syndrome [MIM:612474] | Multiple genes | yes |
| WC4 | 1q21.3 | loss | 0.517 | Mental retardation, autosomal dominant 18 [MIM:614998] | *GATAD2B* | yes |
| WC5 | 4p14 | loss | 0.004 | Nephronophthisis 13 [MIM:614377], AR | *WDR19* | no |
| WC6 | 4p15.31p15.2 | loss | 5.361 | Wolf-Hirschhorn syndrome (PMID: 18932124) | Multiple genes | yes |
| WC7 | 7q11.23 | loss | 1.595 | Williams–Beuren syndrome  [MIM:194050] | Multiple genes | yes |
| WC8 | 7q11.23 | loss | 3.294 | Williams-Beuren syndrome [MIM: 194050]; neurodevelopmental disorder  [PMID 21109226] | *HIP1, YWHAG,* multiple genes | yes |
| WC9 | 11q24.2q25 | loss | 9.458 | Jacobsen syndrome  [JBS, OMIM:147791] | Multiple genes | yes |
| WC10 | 16p11.2 | loss | 1.157 | Idiopathic autism [PMID 18184952, 18156158], developmental delay, and seizures [PMID:19914906]. | Multiple genes | yes |
| WC11 | 16p12.2 | loss | 0.464 | Neurodevelopmental phenotypes  [PMID:20154674] | Multiple genes | yes |
| WC12 | 16q22.1 | loss | 1.034 | 16q22 deletion syndrome [MIM:614541] | Multiple genes | yes |
| WC13 | 17q12 | loss | 1.434 | 17q12 deletion syndrome [MIM: 614527] | *HNF1A* | yes |
| WC14 | 19q13.42 | homozygous  loss | 0.013 | Nemaline myopathy 5, Amish type  [MIM: 605355]; Cardiomyopathy  [MIM: 115210, 613690, 611880] | *TNNT1, TNNI3* | yes |
| WC15 | 20p12.2 | loss | 1.345 | Alagille syndrome [MIM:118450] and tetralogy of fallot [MIM:187500]. | *JAG1* | yes |
| WC16 | 20q13.32 | loss | 0.002 | Pseudo hypoparathyroidism, type IB  [MIM:603233] | *STX16* | no |
| WC17 | Xq28 | loss | 0.228 | Mental retardation, X-linked, FRAXE type [MIM:309548] | *AFF2* | yes |
| WC18 | Chromosome X | loss | 155 | Turner syndrome | Multiple genes | yes |
| WC19 | 6q25.3 | gain | 0.361 | Coffin-Siris syndrome 1  [MIM:614556] | *ARID1B* | no |
| WC20 | 11q13.1q13.2 | gain | 2.435 | Intellectual disability, congenital heart defects, skeletal problems, and ocular abnormalities [PMID:17632770] | Multiple genes | yes |
| WC21 | 17p13.3 | gain | 0.206 | Ectrodactyly and tibia hemimelia with reduced penetrance  [PMID:22147889,23790188] | *BHLHA9* | no |
| WC22,23 | Xq28 | gain | 0.435 | Mental retardation, X-linked syndromic, Lubs type [MIM:300260] | *MECP2* | no |
| WC24* | 13q12.11 | loss | 3.310 | Neurodevelopmental disorder [PMID 25506395] | Multiple genes | yes |
| WC24* | 22q11.1q11.21 | gain | 3.022 | Microduplication 22q11.2 syndrome [MIM:608363] | Multiple genes | yes |
| WC25* | 16p13.11 | gain | 1.166 | Autism [PMID: 17480035] and schizophrenia [PMID:19786961] | Multiple genes | no |
| WC25* | 14q32.2q32.31 | gain | 2.110 | Distal 14q partial duplication [PMID: 27499811] | Multiple genes | no |
| WC26 | 22q13.31 | gain | 2.519 | Microduplication 22q11.2 syndrome [MIM:608363] | Multiple genes | yes |
| WC27 | 20q13.12 | loss | 0.596 | Severe combined immunodeficiency due to ADA deficiency [MIM:102700] | *ADA* | no |

Note: * Patients 24 and 25 each had two CNVs.
